# Rh-CSF1 Attenuates Oxidative Stress and Neuronal Apoptosis via the CSF1R/PLCG2/PKA/UCP2 Signaling Pathway in a Rat Model of Neonatal HIE

**DOI:** 10.1155/2020/6801587

**Published:** 2020-10-07

**Authors:** Xiao Hu, Shirong Li, Desislava Met Doycheva, Lei Huang, Cameron Lenahan, Rui Liu, Juan Huang, Ling Gao, Jiping Tang, Gang Zuo, John H. Zhang

**Affiliations:** ^1^Department of Neurology, Guizhou Provincial People's Hospital, Guiyang 550002, China; ^2^Department of Physiology and Pharmacology, Loma Linda University, Loma Linda, CA 92350, USA; ^3^Department of Neurosurgery, Loma Linda University, Loma Linda, CA 92350, USA; ^4^Burrell College of Osteopathic Medicine, Las Cruces, NM 88003, USA; ^5^Institute of Neuroscience, Chongqing Medical University, Chongqing 400016, China; ^6^Department of Neurosurgery, Affiliated Haikou Hospital of Xiangya Medical College, Central South University, Haikou, Hainan 570208, China; ^7^Department of Neurosurgery, Taicang Hospital Affiliated to Soochow University, Taicang, Suzhou, Jiangsu 215400, China; ^8^Department of Anesthesiology, Loma Linda University, Loma Linda, CA 92350, USA

## Abstract

Oxidative stress (OS) and neuronal apoptosis are major pathological processes after hypoxic-ischemic encephalopathy (HIE). Colony stimulating factor 1 (CSF1), binding to CSF1 receptor (CSF1R), has been shown to reduce neuronal loss after hypoxic-ischemia- (HI-) induced brain injury. In the present study, we hypothesized that CSF1 could alleviate OS-induced neuronal degeneration and apoptosis through the CSF1R/PLCG2/PKA/UCP2 signaling pathway in a rat model of HI. A total of 127 ten-day old Sprague Dawley rat pups were used. HI was induced by right common carotid artery ligation with subsequent exposure to hypoxia for 2.5 h. Exogenous recombinant human CSF1 (rh-CSF1) was administered intranasally at 1 h and 24 h after HI. The CSF1R inhibitor, BLZ945, or phospholipase C-gamma 2 (PLCG2) inhibitor, U73122, was injected intraperitoneally at 1 h before HI induction. Brain infarct volume measurement, cliff avoidance test, righting reflex test, double immunofluorescence staining, western blot assessment, 8-OHdG and MitoSOX staining, Fluoro-Jade C staining, and TUNEL staining were used. Our results indicated that the expressions of endogenous CSF1, CSF1R, p-CSF1R, p-PLCG2, p-PKA, and uncoupling protein2 (UCP2) were increased after HI. CSF1 and CSF1R were expressed in neurons and astrocytes. Rh-CSF1 treatment significantly attenuated neurological deficits, infarct volume, OS, neuronal apoptosis, and degeneration at 48 h after HI. Moreover, activation of CSF1R by rh-CSF1 significantly increased the brain tissue expressions of p-PLCG2, p-PKA, UCP2, and Bcl2/Bax ratio, but reduced the expression of cleaved caspase-3. The neuroprotective effects of rh-CSF1 were abolished by BLZ945 or U73122. These results suggested that rh-CSF1 treatment attenuated OS-induced neuronal degeneration and apoptosis after HI, at least in part, through the CSF1R/PLCG2/PKA/UCP2 signaling pathway. Rh-CSF1 may serve as therapeutic strategy against brain damage in patients with HIE.

## 1. Background

Neonatal hypoxic-ischemic encephalopathy (HIE) is a devastating disease with high morbidity and mortality. It is the primary cause of poor outcomes in infants and leads to lifelong neurodevelopmental disorders, such as cerebral palsy, cognitive deficits, visual dysfunction, hearing impairments, and epilepsy [1–3]. Currently, there is still a lack of effective treatments for HIE.

Over the decades, researchers have delineated many pathological mechanisms that contribute to brain injury, such as inflammation, oxidative stress (OS), blood–brain barrier dysfunction, and apoptosis [4]. OS was one of the main pathologic events in the pathogenesis of numerous neurological diseases [5, 6]. Reactive oxygen species (ROS) are produced as toxic byproducts of aerobic metabolism. The production and detoxification of ROS are tightly balanced under normal circumstances. OS occur when the generation of ROS exceeds the capacity of the antioxidant clearance systems, which is considered as an important pathological factor leading to aging and disease [7]. Previous studies suggested that ROS induced apoptosis by increasing p53 and cytochrome c release, reducing Bcl2, and activating caspase-9 and caspase-3 [8, 9]. After ischemic brain injury, the overproduction of ROS not only directly damaged neurons and resulted in neuronal apoptosis but also indirectly caused neuronal degeneration by regulating mitochondrial pathways, DNA repair enzymes, and transcription factors [10]. Among many factors involved in the pathogenesis of neonatal HIE [11], OS plays a crucial role in the neuronal degeneration and apoptosis [12–14].

CSF1 is produced by a variety of cell types, including monocytes/macrophages, endothelial cells, fibroblasts, and bone marrow stromal cells [15]. CSF1 primarily regulates the proliferation, differentiation, and survival of mononuclear phagocytic systems (e.g., bone marrow progenitors, circulating monocytes, and tissue macrophages) [16], which are mediated by the protein tyrosine kinase receptor, CSF1R [17]. In the central nervous system (CNS), CSF1 is widely expressed in neurons, microglia, astrocytes, and oligodendrocytes [18]. CSF1R is expressed on microglia and hippocampal/cortical neurons under physiological conditions [17]. CSF1R is expressed in immature neurons during early postnatal development, which is gradually reduced in the adult brain [19]. In nestin-positive neural progenitors, ablation of the CSF1R gene resulted in smaller brain volumes, enlarged pools of neural progenitors, and elevated apoptosis in the cortical forebrain [19]. Moreover, the expressions of CSF1 and CSF1R in neurons profoundly increased after a variety of brain injuries [17, 20, 21]. Previous publications mainly focused on the regulation of neuroinflammation by CSF1, but recent studies have reported its protective effects on neurons. The deletion of CSF1R exacerbated cell death and diminished neuronal survival in excitotoxin-induced brain injury [22]. CSF1 was found to improve the survival rate of purkinje cells and reduce neuronal apoptosis in vitro [23]. Intraperitoneal injection of rh-CSF1-containing microcapsules improved the survival rate of neurons in animal models of ischemic brain injury [24]. Wang et al. found that the peripheral administration of CSF1 reduced apoptotic neurons and increased survival rate of neurons in the stroke model, independent on microglia [17, 21]. However, the specific molecular biological mechanism underlying the protection of CSF1 on neurons has not been reported.

CSF1 could activate multiple signaling pathways after binding to CSF1R. Phospholipase C gamma 2 (PLCG2) is a downstream molecule of CSF1R that is activated through phosphorylation. Studies showed that PLCG2 phosphorylation induced by the CSF1R activation could increase the rapid release of Ca^2+^ in monocytes and promote the differentiation of monocytes [25]. PLCG2 is expressed on microglia and neurons in the CNS and is involved in regulating OS [26, 27]. PLCG2 activates the cyclic adenosine monophosphate- (cAMP-) response element-binding protein through activation of the Ca^2+^ signaling pathway induced by protein kinase A (PKA) [28, 29]. The cAMP-PKA signaling pathway is a critical second messenger, which affects a variety of intracellular signal transduction pathways, and regulates protein activity and cell function [30]. It has a significant effect on the metabolism, growth, differentiation, and apoptosis of cells. The phosphorylation of PKA increased after hypoxia and ischemic brain injury [31, 32]. The activation of PKA in the peri-ischemic area played an essential neuroprotective effect in the ischemic stroke model [31, 33, 34] by regulating the function of astrocytes, restoring mitochondrial dynamics, and reducing OS [35, 36]. Uncoupling protein-2 (UCP2) is a mitochondrial endometrial protein that is capable of regulating mitochondrial energy metabolism, decreasing ROS production [37, 38], and alleviating the neurological impairment and neuronal apoptosis after brain trauma and stroke [39]. Activated PKA improved the expression levels of UCP2 and suppressed OS and neuronal apoptosis after subarachnoid hemorrhage in rats [40].

**Figure 1:**
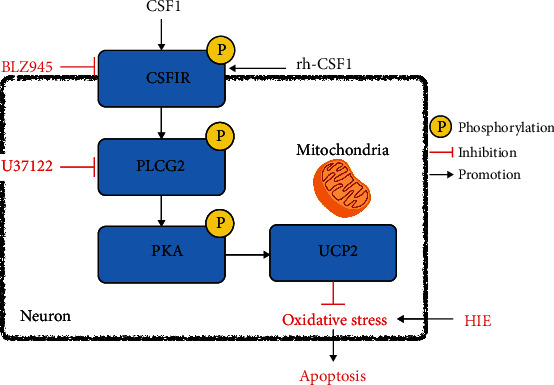
Proposed pathway underlying rh-CSF1 provided neuroprotection. Intranasal administration of rh-CSF1 attenuates OS-induced neuronal degeneration and apoptosis via the CSF1R/PLCG2/PKA/UCP2 signaling pathway in a neonatal rat model of HI.

We previously demonstrated the antineuroinflammation effects of rh-CSF1 after HI [41]. In the current study, we focus on the neuronal protective effect and hypothesize that CSF1 attenuates OS-induced neuronal degeneration and apoptosis through the CSF1R/PLCG2/PKA/UCP2 signaling pathway after HI (shown in Figure 1).

## 2. Materials and Methods

### 2.1. Animals

All experiments were approved by the Institutional Animal Care and Use Committee (IACUC) of Loma Linda University. All studies were conducted in accordance with the National Institutes of Health Guide for the Care and Use of Laboratory Animals. Sprague Dawley rat mothers with litters of 10-12 pups were purchased from Envigo Labs (Livermore, CA). Ten-day old rat pups were used and were housed in an environment with controlled humidity, constant (25°C) temperature with regular 12 h/12 h light/dark cycle and given libitum access to water and food. A total of 127 rat pups (weight = 16 − 22 g) were used in this study, regardless of gender, and were randomly subjected to either sham (*n* = 16) or HI surgery (*n* = 111). However, 7 of 111 HI rats were excluded from the study because of death either during or after hypoxia. All animal tests were conducted blindly.

### 2.2. Experimental Design

#### 2.2.1. Experiment I

To determine the temporal expressions of endogenous CSF1, CSF1R, PLCG2, PKA, and UCP2 at 6 h, 12 h, 24 h, 48 h, 72 h, and 7 d after HI, the rat pups were randomly divided into 7 groups: sham, 6 h HI, 12 h HI, 24 h HI, 48 h HI, 72 h HI, and 7 d HI (*n* = 6/group). Brain samples of the right (ipsilateral) hemisphere were collected for western blot to evaluate the protein expression levels at various time points after HI. The rats in the sham group were sacrificed at 24 h after surgery.

#### 2.2.2. Experiment II

The optimal dose (80 *μ*g/kg) of intranasal administration of rh-CSF1 was selected based on our previous study [41]. Double immunofluorescence staining was used to evaluate the CSF1R expression on neurons and astrocytes at 48 h after HI. To evaluate OS in the HI rat brain, 8-hydroxy-2′-deoxyguanosine (8-OHdG) and MitoSOX staining were conducted at 48 h after HI. To evaluate neuronal degeneration and apoptosis in the HI rat brain, Fluoro-Jade C (FJC) staining and terminal deoxynucleotidyl transferase dUTP nick end labeling (TUNEL) staining were conducted. Rat pups were randomized into 3 groups (*n* = 4/group): sham, HI+vehicle (vehicle of rh-CSF1 and DDH_2_O), and HI+rh-CSF1. All three groups were sacrificed at 48 h after surgery.

#### 2.2.3. Experiment III

To investigate the underlying mechanisms of anti-OS and the antiapoptotic effects of rh-CSF1, BLZ945 (inhibitor of CSF1R) and U73122 (inhibitor of PLCG2) were used. The groups included sham, HI+vehicle (vehicle of rh-CSF1 and DDH_2_O), HI+rh-CSF1, HI+rh-CSF1+DMSO (vehicle of BLZ945 and U73122), HI+rh-CSF1+BLZ945, and HI+rh-CSF1+U73122 with the *n* = 10/group, of which 6 pups for 2,3,5-triphenyltetrazolium chloride monohydrate (TTC) (Sigma Aldrich Inc., USA) staining and western blot and 4 pups for immunofluorescence staining. Short-term neurobehavioral tests (cliff avoidance test and righting reflex test), TTC staining, western blot, FJC, TUNEL, 8-OHdG staining, and MitoSOX staining were conducted at 48 h after HI. All six groups were sacrificed at 48 h after surgery.

#### 2.2.4. Experiment IV

To investigate the efficiency of CSF1R and PLCG2 inhibition and the endogenous neuroprotective mechanism of the CSF1/CSF1R/PLCG2/PKA/UCP2 signaling pathway in this study, BLZ945 and U73122 were used. The groups included HI+DMSO (vehicle of BLZ945 and U73122), HI+BLZ945, and HI+U73122 groups (*n* = 6/group). The right (ipsilateral) hemisphere of brain tissues were collected for western blot to evaluate expression levels of CSF1, CSF1R, p-CSF1R, p-PLCG2, p-PKA, UCP2, Bcl2, Bax and Cleaved caspase-3. All three groups were sacrificed at 48 h after surgery.

### 2.3. HI Model

The animal model of neonatal HI was performed as previously described [42]. Briefly, the rat pups were anesthetized using 3% isoflurane and maintained with 2.5% isoflurane during surgery. A longitudinal midline neck incision was made on the right anterior neck, and the right common carotid artery was identified, isolated, and double ligated with 5.0 surgical silk surgical suture. The artery was then cut between the ligations, and the duration of the procedure was limited to 5–9 minutes. After the surgical procedure, the rat pups were given time to recover from anesthesia for 1 h on temperature-controlled heating blankets. Pups were then subjected to 2.5 h of hypoxia in a chamber with 8% O_2_ and 92% N_2_ in a 37°C water bath. For the sham treatment, the right common carotid artery was exposed, but without ligation or exposure to hypoxic conditions. After hypoxia, the animals were returned to their mother and left in the incubator for 48 h.

### 2.4. Drug Administration

Rat pups were placed in a supine position under 2% isoflurane anesthesia at 1 h and 24 h after HI, and a total volume of 5 *μ*l rh-CSF1 (80 *μ*g/kg, Abcam, USA) or vehicle (DDH_2_O) was delivered via intranasal route with a drop of 1.25 *μ*l given every 2 min, alternating between the left and right nares. BLZ945 (60 mg/kg, Cayman chemical, USA), U73122 (30 mg/kg, Cayman chemical, USA), or vehicle of BLZ945 and U73122 (10% DMSO dissolved in corn oil) was delivered via intraperitoneal injection, based on previous studies at 1 h prior to HI induction [39].

### 2.5. Neurological Evaluation

At 48 h after HI, short-term neurobehavior tests including the cliff avoidance and righting reflex tests were conducted by an investigator blinded to group information.

#### 2.5.1. Cliff Avoidance Test

Cliff avoidance tests were conducted by placing rat pups on the edge of the platform (30 cm × 30 cm × 30 cm), with the forepaws and chest extending over the edge. The latency of the rats to turn away or retreat from the edge was recorded. If the rats fell from the platform, or if they did not respond within 60 s, the latency was recorded as 60 s.

#### 2.5.2. Righting Reflex Test

The righting reflex test is used to record the duration it took for the pups to completely roll over onto four limbs after being placed in the supine position. The maximum testing time was 60 s, and any recordings exceeding 60 s were documented as 60 s (3 trials/pup/day). Lastly, the average values of all three trials were calculated.

### 2.6. Infract Volume Measurement

After neurological testing at 48 h after HI, the rat pups were transcardially perfused with 20 ml prechilled PBS under deep anesthesia. The brains were cut into 2 mm thick coronal sections. The brain sections were incubated in 2% TTC (Sigma Aldrich Inc., USA) solution for 5 min in the dark and then rinsed with PBS [43]. Brain infarct volume was quantified and analyzed using ImageJ software (NIH, USA). The percentage of infarcted areas was calculated as follows: [(total area of contralateral hemisphere)-(area of uninfarcted area of ipsilateral hemisphere)]/(total area of contralateral hemisphere×2).

### 2.7. Western Blotting Analysis

Western blot tests were performed as previously described [44]. After TTC staining and digitally photographing at 48 h after HI, the brain sections were divided into ipsilateral and contralateral hemispheres, snap frozen in the liquid nitrogen, and stored in a −80°C freezer until further tissue lysis. The ipsilateral hemisphere samples were extracted in RIPA lysis buffer (Santa Cruz Biotechnology, USA) and a protease inhibitor cocktail for 15 min and then further centrifuged at 14,000 g at 4°C for 30 min. The protein concentration was determined by collecting the supernatant and using a detergent compatibility assay (Bio-Rad, DC™ Protein Assay). Equal amounts of protein were loaded onto each lane of 10% sodium dodecyl sulfate–polyacrylamide gel. After electrophoresis, the proteins were transferred onto nitrocellulose membranes, which were blocked with 5% nonfat blocking grade milk (Bio-Rad, Hercules, USA) and incubated with the primary antibodies overnight at 4°C. The following primary antibodies were used: anti-CSF1 (1 : 1000, Abcam, USA), anti-CSF1R (1 : 500, LSBio, USA), anti-p-CSF1R (1 : 1000, Thermo Fisher Scientific, USA), anti-PLCG2 (1 : 500, Novus biologicals, USA), anti-p-PLCG2 (1 : 1000, Abcam, USA), anti-PKA (1 : 1000, Abcam, USA), anti-p-PKA (1 : 1000, Abcam, USA), anti-UCP2 (1 : 1000, Cell Signaling Technology. USA), anti-Bcl2 (1 : 1000, Abcam, USA), anti-Bax (1 : 500, Abcam, USA), anti-cleaved caspase-3 (1 : 500, Cell Signaling Technology, USA), and Goat anti-*β*-actin (1 : 3000, Santa Cruz Biotechnology, USA). On the following day, the membranes were incubated with the appropriate secondary antibodies (1 : 3000, Santa Cruz Biotechnology, USA) at room temperature for 2 h. The optical densities of the bands were then visualized with the ECL Plus chemiluminescence reagent kit (Amersham Biosciences, USA) and were analyzed using Image J software (NIH, USA).

### 2.8. Histology and Immunohistochemistry

Rat pups were transcardially perfused with ice cold PBS, followed by 10% formalin under deep anesthesia at 48 h after HI. The brains were postfixed in 10% formalin overnight at 4°C for 24 h and then dehydrated in 30% sucrose in PBS solution at 4°C until they sank. Brain samples were frozen at -80°C after embedding in OCT (Scigen Scientific, USA). The coronal slices were cut into 8-10 *μ*m thickness for immunofluorescence staining at -20°C using a cryostat (LM3050S; Leica Microsystems, Germany).

#### 2.8.1. Immunofluorescence Staining

Immunofluorescence staining was routinely performed [45]. The slices were rinsed with PBS for 30 min and then permeabilized with 0.3% Triton X-100 for 10 min at room temperature. The slices were then rinsed with PBS for 15 min and blocked with 5% donkey serum for 2 h at room temperature. Subsequently, each coronal slice was incubated with primary antibodies at 4°C overnight. The following primary antibodies were used: anti-neuronal nuclei (NeuN) (1 : 200 Abcam, USA), anti-glial fibrillary acidic protein (GFAP) (1 : 200, Abcam, USA), anti-CSF1 (1 : 100, Abcam, USA), anti-CSF1R (1 : 100, LSBio, USA), and anti-8-OHdG (1 : 200, Abcam, USA). The following day, the slices were rinsed with PBS for 1 h and then incubated with the corresponding secondary antibodies (1 : 150) for 1 h at room temperature. Finally, the slices were rinsed with PBS for 1 h and then covered with *4*′*,6*-*diamidino-2-phenylindol*e (DAPI) (Vector Laboratories Inc., USA) to show the total nuclei. The stained slices were visualized and photographed under a fluorescence microscope (Leica DMi8, Leica Microsystems, Germany) and analyzed by Leica Application Suite software. Four rat brains per group were counted from the 5 fields per brain within the perilesion area for quantification analysis. The data was presented as the total number of CSF1R-positive NeuN, CSF1R-positive GFAP, CSF1-positive NeuN, and CSF1-positive GFAP cells per square millimeter (cell/mm^2^). The data was presented as the average ratio of 8-OHdG-positive cells (%).

#### 2.8.2. MitoSOX Staining

The mitochondrial ROS level was measured using MitoSOX Red. The brain slices were incubated in the dark 5 *μ*mol/L with MitoSOX (Thermo Fisher Scientific, USA) for 10 min at 37°C and then covered with DAPI for 5 min at room temperature. The stained slices were visualized and photographed under a fluorescence microscope and analyzed using Leica Application Suite software. Four rat brains per group were counted from the 5 fields per brain within the perilesion area for quantification analysis. The data was presented as the average ratio of MitoSOX-positive cells (%).

#### 2.8.3. FJC Staining

To detect degenerating neurons, FJC staining was performed using the FJC Ready-to-Dilute Staining Kit (Biosensis, USA) according to the manufacturer's protocol. The stained slices were visualized and photographed under a fluorescence microscope and analyzed using Leica Application Suite software. Four rat brains per group were counted from the 5 fields per brain within the perilesion area for quantification analysis. The data was presented as the average number of FJC-positive cells per square millimeter (cell/mm^2^).

#### 2.8.4. TUNEL Staining

To detect neuronal apoptosis, double immunofluorescence staining was conducted at 48 h after HI by using TUNEL (red) and neuron marker NeuN (green) with the in situ Apoptosis Detection Kit (Roche, USA) according to the manufacturer's protocol [46]. The stained slices were visualized and photographed under a fluorescence microscope and analyzed by Leica Application Suite software. Four rat brains per group were counted from the 5 fields per brain within the perilesion area for quantification analysis. The data was presented as the average number of TUNEL-positive neurons per square millimeter (cell/mm^2^).

### 2.9. Statistical Analysis

Statistical analysis was completed using SPSS v.21.0 (IBM, USA). Data was presented as means ± SD. Differences between individual groups were first compared using single factor analysis of variance (one-way ANOVA), followed by multiple comparison between groups using Tukey's post hoc analysis. Differences between the two groups were compared using Student's *t*-test. Data were considered statistically significant when *P* < 0.05.

## 3. Results

### 3.1. Time Course Expression Levels of Endogenous Proteins (p-CSF1R, CSF1R, CSF1, p-PLCG2, p-PKA, and UCP2) after HI

**Figure 2:**
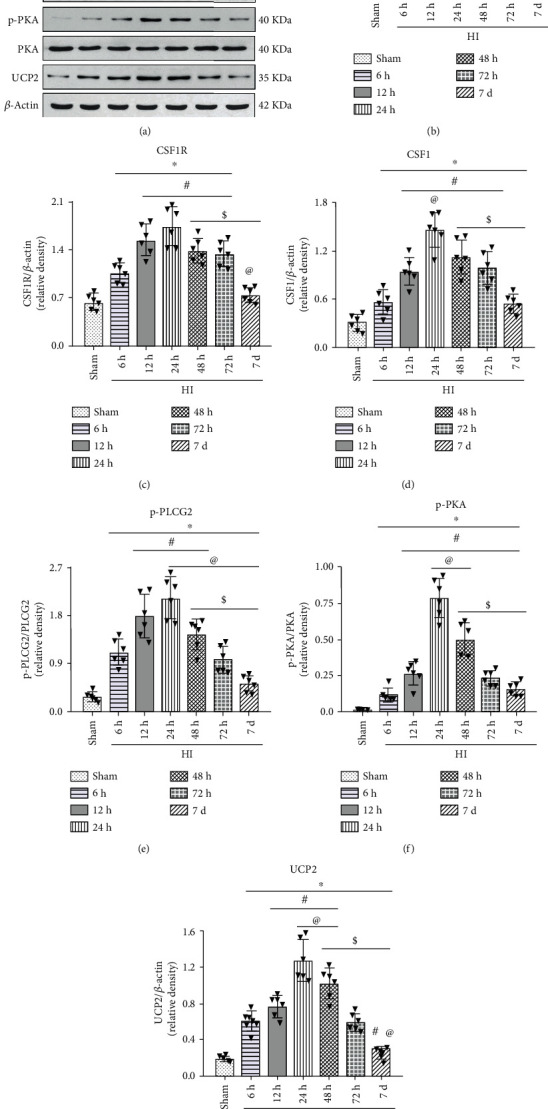
Temporal expression of endogenous p-CSF1R, CSF1R, CSF1, p-PLCG2, p-PKA, and UCP2 in the brain after HI. (a) Representative western blot bands. (b–g) Quantitative analysis of the relative protein levels of p-CSF1R, CSF1R, CSF1, p-PLCG2, p-PKA, and UCP2 after HI insult. ∗P < 0.05 vs sham; ^#^P < 0.05 vs 6 h HI; ^@^P < 0.05 vs 12 h HI; ^$^P < 0.05 vs 24 h HI. n = 6 per group.

The endogenous expression levels of p-CSF1R, CSF1R, CSF1, p-PLCG2, p-PKA, and UCP2 were measured by western blot. The results showed that p-CSF1R, CSF1R, CSF1, p-PLCG2, p-PKA, and UCP2 were increased in a time-dependent manner after HI peaked at 24 h (*P* < 0.05) when compared with the sham group (Figure 2).

### 3.2. The Cellular Expression of CSF1R Receptor and CSF1 after HI

**Figure 3:**
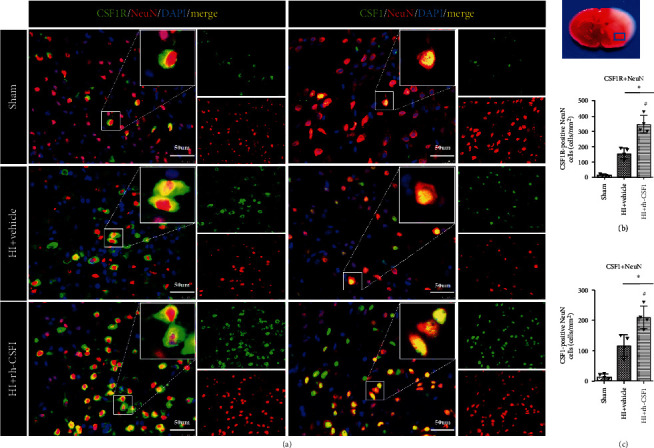
Immunofluorescence staining of CSF1R and CSF1 with NeuN in the brain at 48 h after HI. (a) Representative microphotographs of CSF1R or CSF1 with NeuN staining in the ipsilateral cortex of the sham, HI+vehicle, and HI+rh-CSF1 groups. (b) Quantitative analysis of CSF1R-positive NeuN cells in the ipsilateral cortex at 48 h after HI. (c) Quantitative analysis of CSF1-positive NeuN cells in the ipsilateral cortex at 48 h after HI. Green indicated CSF1R- or CSF1-positive staining, red indicated NeuN-positive neuron staining, and blue indicated DAPI-positive nuclear staining. Merge showed the colocalization of CSF1R or CSF1 with neurons. ^∗^P < 0.05 vs sham; ^#^P < 0.05 vs vehicle; scale bar = 50 *μ*m; n = 4 per group. The upper right panel of the brain slice indicates the location for staining analysis (small blue box).

Double immunofluorescence staining of either the CSF1R receptor or CSF1 with NeuN and GFAP was conducted in the three groups of sham, HI+vehicle, and HI+rh-CSF1. Our results showed that CSF1R and CSF1 were expressed on neurons in rat pups from all groups at 48 h after HI (Figure 3(a)). Although the number of neurons was reduced, CSF1-positive and CSF1R-positive neurons were increased in the HI+vehicle group compared with shams. Intranasal rh-CSF1 treatment attenuated the neuronal loss and further increased CSF1R-positive and CSF1-positive neurons when compared to the HI+vehicle group (Figures 3(b) and 3(c)).

**Figure 4:**
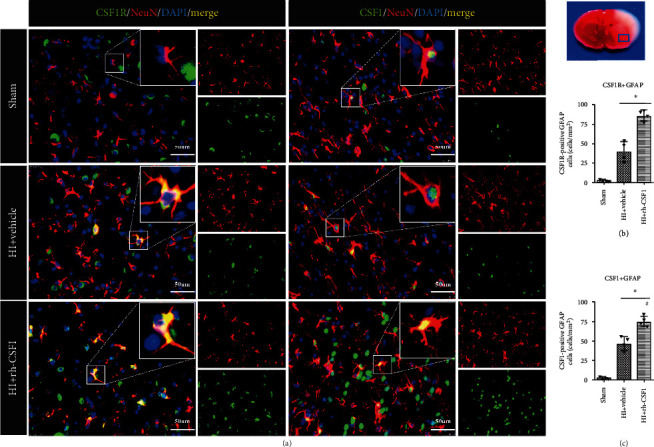
Immunofluorescence staining of CSF1R or CSF1 with GFAP in the brain at 48 h after HI. (a) Representative microphotographs of CSF1R and CSF1 with GFAP staining in the ipsilateral cortex of the sham, HI+vehicle, and HI+rh-CSF1 groups. (b) Quantitative analysis (T test) of CSF1R-positive GFAP cells in the ipsilateral cortex at 48 h after HI. (c) Quantitative analysis of CSF1-positive GFAP cells in the ipsilateral cortex at 48 h after HI. Green indicated CSF1R- or CSF1-positive staining, red indicated GFAP-positive astrocytes staining, and blue indicated DAPI-positive nuclear staining. Merge showed the colocalization of CSF1R or CSF1 with astrocytes. ^∗^P < 0.05 vs sham; ^#^P < 0.05 vs vehicle; scale bar = 50 *μ*m; n = 4 per group. The upper right panel of brain slice indicates the location for staining analysis (small blue box).

At 48 h after HI, CSF1 was expressed on astrocytes in rat pups from all three groups. The astrocyte expression of CSF1R was not found in the shams but in HI rat pups (Figure 4(a)). With increases in the number of astrocytes after HI, CSF1-positive and CSF1R-positive astrocytes were increased compared with the sham group. Intranasal rh-CSF1 treatment reduced the number of astrocytes, but further increased CSF1-positive and CSF1R-positive astrocytes in the HI+rh-CSF1 group when compared to the HI+vehicle group (Figures 4(b) and 4(c)).

### 3.3. Inhibition of CSF1R or PLCG2 Abolished the Neuroprotective Effects of Rh-CSF1 Treatment at 48 H after HI

**Figure 5:**
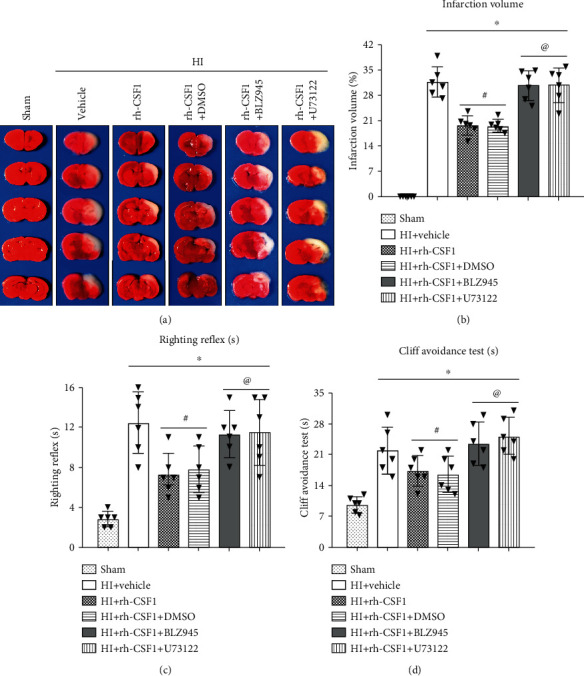
Effects of rh-CSF1, CSF1R, and PLCG2 inhibitors on the infarct volume and neurological function at 48 h after HI. (a) Representative TTC staining photographs. (b) Quantitative analysis of the infarct volume. (c) Cliff avoidance test. (d) Righting reflex test. ^∗^P < 0.05 vs. sham; ^#^P < 0.05 vs. HI+rh-CSF1 vehicle; ^@^P < 0.05 vs. HI+rh-CSF1+DMSO. n = 6 per group.

To determine whether CSF1 exerts its neuroprotective effect via the CSF1R/PLCG2/PKA/UCG2 signaling pathway, BLZ945 (the specific CSF1R inhibitor) and U73122 (the specific PLCG2 inhibitor) were used. TTC staining data showed that intranasal administration of rh-CSF1 markedly reduced the infarct volume of HI animals when compared with the HI+vehicle group (Figures 5(a) and 5(b)). Both BLZ945 and U73122 reversed the beneficial effects of rh-CSF1, characterized by an increase in the infarcted area when compared with the corresponding control groups (*P* < 0.05, Figures 5(a) and 5(b)).

The cliff avoidance test showed that the rat pups spent more time turning away or retreating from the edge after HI when compared with the sham group. The rat pups that received rh-CSF1 had markedly improved performance when compared to the HI+vehicle group (*P* < 0.05, Figure 5(c)). However, BLZ945 or U73122 significantly reversed the effects of rh-CSF1 when compared with each corresponding control group (*P* < 0.05, Figure 5(c)).

The righting reflex test results showed that rat pups required longer time to correct their positioning after HI when compared with the sham group, which was improved by the rh-CSF1 treatment. However, BLZ945 or U73122 significantly reversed the benefits of rh-CSF1 when compared with each corresponding control group (*P* < 0.05, Figure 5(d)).

### 3.4. Rh-CSF1 Administration Suppressed OS, Alleviated Neuronal Degeneration, and Attenuated Neuronal Apoptosis through the CSF1R/PLCG2/PKA/UCP2 Signaling Pathway at 48 H after HI

**Figure 6:**
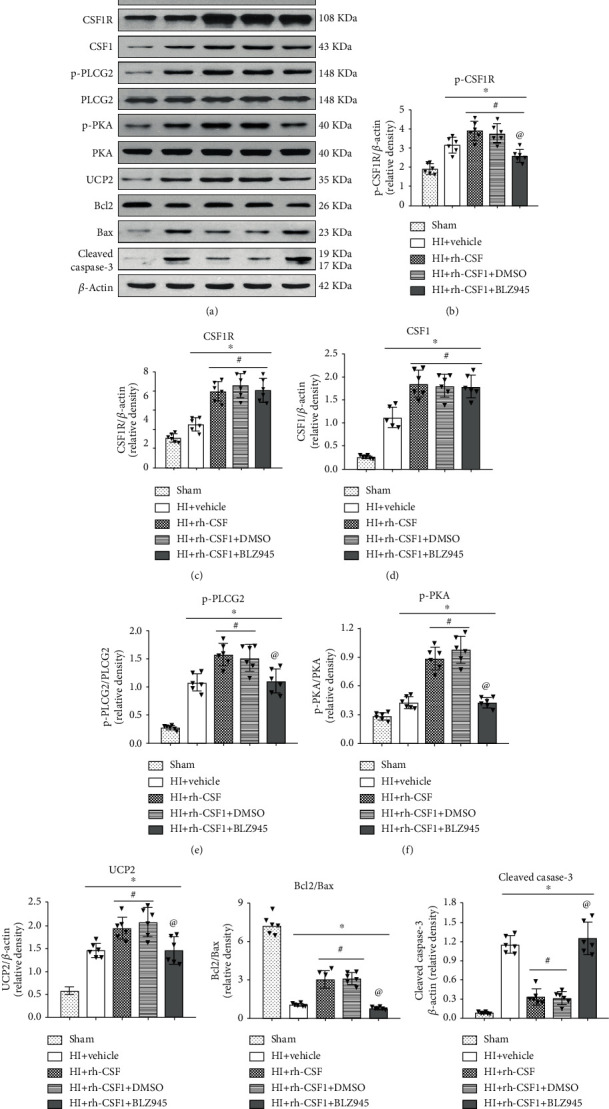
Effects of rh-CSF1 and CSF1R inhibitor on downstream protein expression levels in the proposed signaling pathway with rh-CSF1 treatment at 48 h after HI. (a) Representative western blot bands of p-CSF1R, CSF1R, CSF1, p-PLCG2, p-PKA, UCP2, Bcl2, Bax, and cleaved caspase-3. (b–i) Quantitative analysis of the relative protein levels after HI insult. ^∗^P < 0.05 vs. sham; ^#^P < 0.05 vs. HI+vehicle; ^@^P < 0.05 vs. HI+rh-CSF1+DMSO; n = 6 per group.

**Figure 7:**
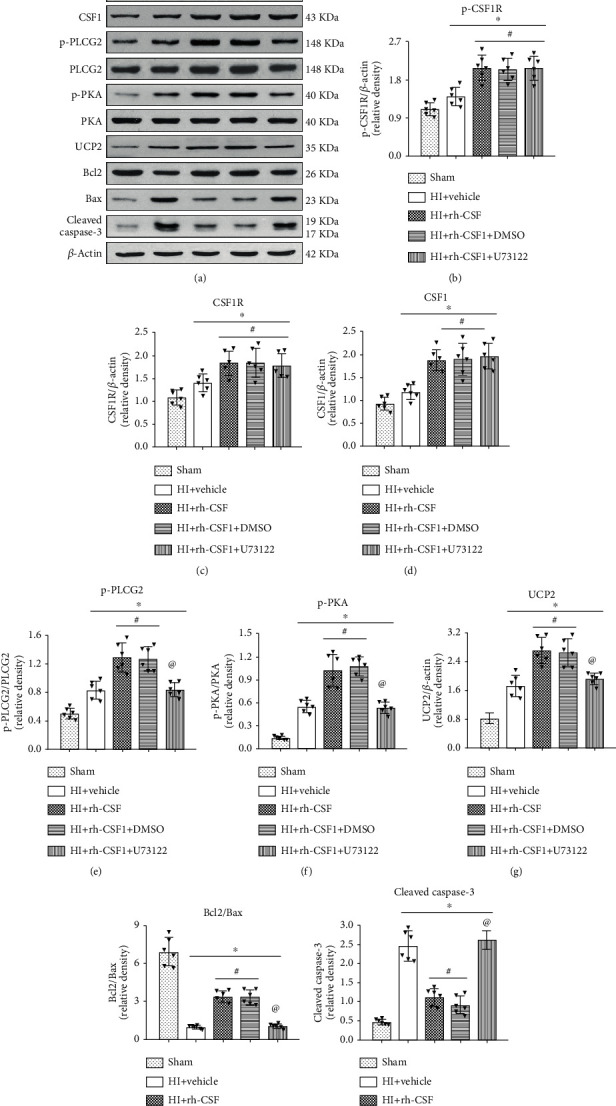
Effects of rh-CSF1 and PLCG2 inhibitor on downstream protein expression levels in the proposed signaling pathway with rh-CSF1 treatment at 48 h after HI. (a) Representative western blot bands of p-CSF1R, CSF1R, CSF1, p-PLCG2, p-PKA, UCP2, Bcl2, Bax, and cleaved caspase-3. (b–i) Quantitative analysis of the relative protein levels after HI insult. ^∗^P < 0.05 vs. sham; ^**#**^P < 0.05 vs. HI+vehicle; ^**@**^P < 0.05 vs. HI+rh-CSF1+DMSO; n = 6 per group.

To determine whether the CSF1R/PLCG2/PKA/UCP2 signaling pathway was involved in the underlying neuroprotective mechanism of CSF1 following HI, the expression levels of the pathway-related proteins were measured by western blot. Compared with the sham group, the expressions of CSF1, total-CSF1R, p-CSF1R, p-PLCG2, p-PKA, UCP2, and cleaved caspase-3 were increased, but the Bcl2/Bax ratio was significantly decreased at 48 h after HI. Rh-CSF1 treatment further increased the expression of CSF1, total-CSF1R, p-CSF1R, p-PLCG2, p-PKA, UCP2 and the ratio of Bcl2/Bax, but reduced the expression of cleaved caspase-3 compared with the HI+vehicle group. The CSF1R inhibitor decreased p-CSF1R expressions and reversed the effects of rh-CSF1 on protein levels of p-PLCG2, p-PKA, UCP2, the ratio of Bcl2/Bax, and the expression of cleaved caspase-3 (*P* < 0.05, Figures 6(a)–6(g)). In the absence of changes in p-CSF1R, the PLCG2 inhibitor decreased the p-PLCG2 expression also reversed the effects of rh-CSF1 on subsequent protein levels of p-PKA, UCP2, the ratio of Bcl2/Bax, and the expression of cleaved caspase-3 (*P* < 0.05, Figures 7(a)–7(g)).

**Figure 8:**
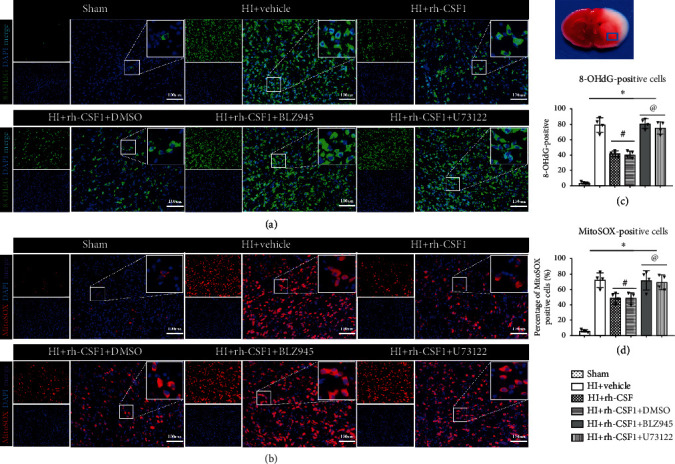
Effects of rh-CSF1 on mitochondria OS after HI at 48 h after HI. (a) Representative microphotographs of 8-OHdG staining in the ipsilateral cortex of the rat brain. (b) Representative microphotographs of MitoSOX staining in the ipsilateral cortex of the rat brain. (c) Quantitative analysis of 8-OHdG-positive cells in the ipsilateral cortex. (d) Quantitative analysis of MitoSOX-positive cells in the ipsilateral cortex 48 h after HI. ^∗^P < 0.05 vs. sham; ^**#**^P < 0.05 vs. HI+vehicle; ^**@**^P < 0.05 vs. HI+rh-CSF1+DMSO; scale bar = 100 *μ*m; n = 4 per group. The upper right panel of the brain slice indicates the location for staining analysis (small blue box).

**Figure 9:**
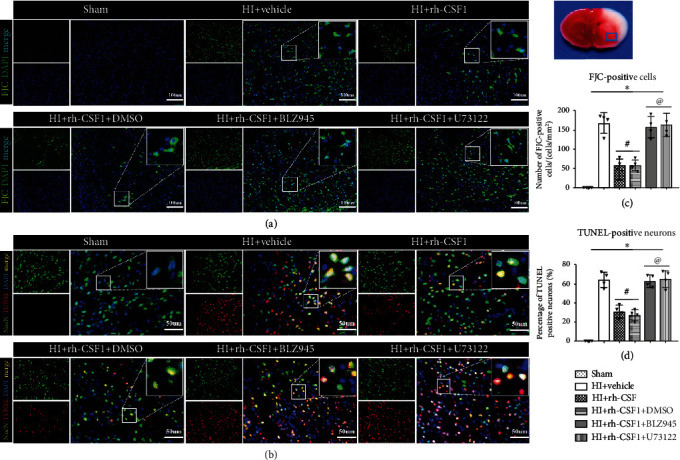
Effects of rh-CSF1 on neuronal degeneration and apoptosis after HI at 48 h after HI. (a) Representative microphotographs of FJC staining in the ipsilateral cortex of the rat brain. (b) Representative microphotographs of TUNEL staining in the ipsilateral cortex of the rat brain. (c) Quantitative analysis of FJC-positive cells in the ipsilateral cortex. (d) Quantitative analysis of TUNEL-positive neurons in the ipsilateral cortex. ^∗^P < 0.05 vs. sham; ^#^P < 0.05 vs. HI+vehicle; ^**@**^P < 0.05 vs. HI+rh-CSF1+DMSO; scale bar = 100 *μ*m (FJC staining); scale bar = 50 *μ*m (TUNEL staining), n = 4 per group. The upper right panel of brain slice indicates the location for staining analysis (small blue box).

To further determine whether rh-CSF1 treatment suppressed OS, alleviated neuronal degeneration, and attenuated neuronal apoptosis through the CSF1R/PLCG2/PKA/UCP2 signaling pathway, MitoSOX staining, 8-OHdG staining, FJC staining, and TUNEL staining were conducted at 48 h after HI. When compared with the sham group, MitoSOX-, 8-OHdG-, and FJC-positive cells and TUNEL-positive neurons were increased at 48 h after HI, which was attenuated by the intranasal administration of rh-CSF1 (*P* < 0.05, Figures 8 and 9(a)–9(d)). CSF1R or PLCG2 inhibitor abolished the protective effects of rh-CSF1, as evidenced by the increased number of MitoSOX-, 8-OHdG-, and FJC-positive cells and TUNEL-positive neurons when compared with corresponding control groups (*P* < 0.05, Figures 8 and 9(a)–9(d)). Collectively, these results suggest that rh-CSF1 treatment suppressed OS and improved neuronal survival through the CSF1R/PLCG2/PKA/UCP2 signaling pathway after HI.

### 3.5. The Effects of CSF1R and PLCG2 Inhibitor on Pathway-Related Protein Expression Levels at 48 H after HI

**Figure 10:**
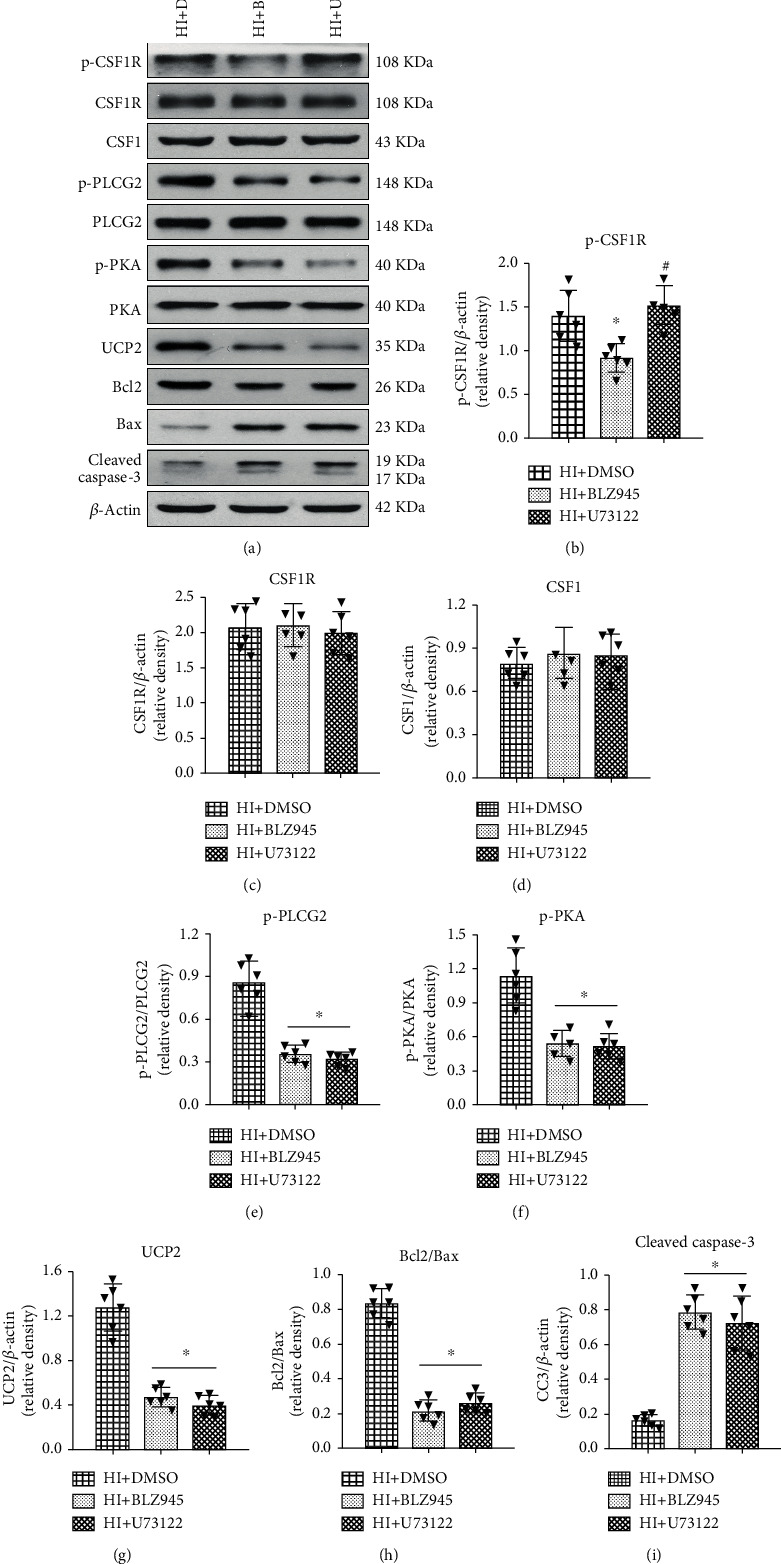
Effects of CSF1R and PLCG2 inhibitor on pathway-related protein expression levels at 48 h after HI. (a) Representative western blot bands of p-CSF1R, CSF1R, CSF1, p-PLCG2, p-PKA, UCP2, Bcl2, Bax, and cleaved caspase-3. (b–i) Quantitative analysis of the relative protein levels at 48 h after HI. Data are represented as means ± SD. ^∗^P < 0.05 vs. HI+DMSO; ^**#**^P < 0.05 vs. HI+BLZ945. n = 6 for each group.

Western blot was conducted at 48 h after HI in HI+DMSO (vehicle of BLZ945 and U73122), HI+BLZ945, and HI+U73122 groups. Inhibition of CSF1R and PLCG2 decreased the expression of p-CSF1R and p-PLCG2, accompanied by reduction of p-PKA, UCP2, and Bcl2, but increased the apoptotic-associated proteins Bax and cleaved caspase-3 (Figure 10). The results demonstrated the efficiency of CSF1R and PLCG2 inhibition in this study and also showed that the CSF1/CSF1R/PLCG2/PKA/UCP2 signaling pathway played an endogenous neuroprotective role after HI.

## 4. Discussion

In the present study, we focused on the effects of rh-CSF1 treatment in reducing OS-induced neuronal degeneration and apoptosis following HI injury. Our results showed that (1) following HI, the expressions of proteins in the CSF1R/PLCG2/PKA/UCP2 signaling pathway were increased in a time-dependent manner and peaked at 24 h after HI. (2) HI caused OS injury (evidence by 8-OHdG and MitoSOX-positive staining), significant neuronal degeneration (evidenced by FJC positive staining), and neuronal apoptosis (evidenced by TUNEL-positive staining, the reduced Bcl2/Bax ratio, and increased cleaved caspase-3 level in the brain). (3) Intranasal administration of exogenous rh-CSF1 reduced the infarct area, improve short-term neurological deficits, and upregulated the protein levels of the CSF1R/PLCG2/PKA/UCP2 signaling pathway. (4) CSF1R and PLCG2 inhibitors reversed the neuroprotective effects of rh-CSF1 as well as its effects on protein levels of the CSF1R/PLCG2/PKA/UCP2 signaling pathway. These results suggested that rh-CSF1 is neuroprotective against OS-induced neuronal degeneration and apoptosis following HI. Such protection is, at least in part, through the CSF1R/PLCG2/PKA/UCP2 signaling pathway.

CSF1 is a single-pass type I membrane cytokine and has an essential role in regulating the survival, proliferation, and differentiation of hematopoietic precursor cells, especially mononuclear phagocytes, such as macrophages and monocytes [16, 47]. In the CNS, CSF1 contributes to the proliferation and differentiation of microglia and is critical to microglia development [48]. Therefore, CSF1 is involved in the immune and inflammatory responses of various CNS diseases, including experimental autoimmune encephalomyelitis [49], Alzheimer's disease (AD) [18], Parkinson's disease [50], and stroke [51]. Through its receptor CSF1R, CSF1 plays a biological role [17]. Our previous study demonstrated that CSF1 and its receptor were expressed on microglia and rh-CSF1 treatment attenuated the neuroinflammation in a rat model of HIE [41]. However, CSF1R is not only expressed in microglia but also in neurons. The expression levels of CSF1 and CSF1R on neurons were significantly upregulated after various neurological diseases [17, 20, 52]. CSF1 directly reduced neuronal apoptosis after AD [17]. In the present study, we found that CSF1 and CSF1R colocalized with neurons, and there were increased numbers of CSF1 and CSF1R-positive neurons after HI. Interestingly, CSF1 and CSF1R also colocalized with astrocytes, and HI insult induced the increases in numbers of CSF1 and CSF1R-positive astrocytes as well. As important inflammatory regulators, astrocytes produce anti-inflammatory and neurotrophic factors, release numerous chemokines to adjust the microenvironment of brain [53, 54], and secrete antioxidant substrates, such as glutathione to alleviate neuronal damage [55]. However, excessive astrocytes activations could aggravate neuronal damage by releasing many proinflammatory factors, such as TNF-*α* and IL-6 [56]. Wylot et al. reported that CSF1 provided a significant effect in maintaining the balance between microglia and astrocytes [57]. Thus, the increased endogenous CSF1 and CSF1R expressions on neurons and astrocytes may implicate the upregulation of an endogenous neuroprotective mechanism after HI. In ipsilateral brain tissue, endogenous protein levels of CSF1, CSF1R, PLCG2, PKA, and UCP2 were increased overtime after HI, suggesting that they play a role after HI. Inhibitor of CSF1R and PLCG2 decreased the expression of p-CSF1R and p-PLCG2, accompanied by reduction of p-PKA and UCP2 after HI. The results demonstrated that the CSF1/CSF1R/PLCG2/PKA/UCP2 signaling pathway played an endogenous neuroprotective role, but the effects was not sufficient to improve the neuronal damage after HI. Exogenous treatment of rh-CSF1 might upregulate the expression of CSF1 and further activate the signaling pathway to provide neuroprotective effects after HIE.

The intranasal administration of rh-CSF1 further enhanced the elevation of CSF1 and CSF1R on neurons and astrocytes, leading to improved short-term neurological outcomes with significantly less infarction volumes, neuronal degeneration, and apoptosis. Our findings echoed other studies showing that CSF1 reduced neuronal damage after stroke, experimental autoimmune encephalomyelitis, and AD [16, 21, 22]. Neurons were more vulnerable to ischemia and chemical damage in CSF1-deficient mice [58]. Following HI, OS was significantly increased which are associated with neuroinflammation, mitochondrial dysfunction, glial/axonal mutagenesis, and synaptic transmission disorders, all contributing to progressive loss of neurons including neuronal apoptosis [13, 59]. Our study confirmed that there are increased cellular OS markers MitoSOX and 8-OHdG associated with more FJC-/TUNEL-positive degenerated and apoptotic neurons within the perilesion brain regions of HI rat pups. Meanwhile, protein expression levels of cleaved caspase-3 were increased, but the ratio of Bcl2/Bax was decreased in brain tissues. Intranasal rh-CSF1 treatment significantly attenuated OS markers and neuron damages, leading to an improved short-term neurobehavioral outcome. We further elucidated the signaling pathway of CSF1/CSF1R/PLCG2/UCP2 underlying the neuronal protection effects using CSF1R inhibitor and PLCG2 inhibitor.

In our experiment, rh-CSF1 increased the expression levels of CSF1, CSF1R, phosphorylation of PLCG2, and UCP2. BLZ945 (the specific inhibitor of CSF1R) or U73122 (PLCG2 inhibitor) reversed the effects of rh-CSF1 on CSF1/CSF1R/PLCG2/UCP2 signaling. These two inhibitors also abolished the neuroprotective effects of rh-CSF1. PLCG2, as one of classic downstream proteins of CSF1R, can interact with CSF1R to induce the release of Ca^2+^ [25, 60]. U73122 inhibited phosphorylation of PLCG2 on Thr172, which attenuated the rapid release of Ca^2+^ triggered by CSF1 in human monocytes [25]. In the CNS, PLCG2 was mainly expressed on microglia, but was also expressed on neurons in the granulosa cell layer of the dentate gyrus [61]. PLCG2 is involved in alleviating ROS levels and is closely associated with OS [26, 27]. Other studies found that PLCG2 regulated mTOR through the diacylglycerol/protein kinase C signaling branch, thereby affecting the proliferation and apoptosis of B cells [62]. PKA is cAMP-dependent protein kinase, which played a crucial role in inducing cell signal response and regulating the signal transduction pathway. Previous studies showed that PKA provided important neuroprotective effects and reduced neuronal apoptosis through the p38 MAPK/CREB or CREB/BDNF signaling pathway in various CNS conditions, including ischemic brain injury, Parkinson's disease, and AD [63–66]. PLCG2 could activate PKA and promote the phosphorylation of PKA [28, 29]. Mo et al. showed that PKA exerted an antiapoptotic role by activating UCP2 after subarachnoid hemorrhage [40]. UCP2 can provide an important effect in attenuating mitochondrial ROS levels and cell death [67, 68]. Increased expression or activation of UCP2 could suppress neuronal damage induced by OS in CNS diseases, such as epilepsy, stroke, and subarachnoid hemorrhage [36, 37, 58]. UCP2 also exerted an essential neuroprotective role after HI by preventing neuronal apoptosis via OS inhibition [12]. In the present study, we found that activation of CSF1R with rh-CSF1 increased the phosphorylation of PKA and the expression of UCP2 in the rat brain at 48 h after HI, accompanied by the improvement of neurological dysfunctions, and inhibition of OS-induced neuronal degeneration and apoptosis. Our results implied that rh-CSF1 reduced OS-induced neuronal degeneration and apoptosis after HI, at least in part, through the CSF1R/PLCG2/PKA/UCP2 signaling pathway.

There are some limitations in this study. First, CSF1 was also expressed on astrocytes and microglia. We cannot exclude the CSF1/CSF1R signaling of astrocytes and microglial in contribution to the neuronal protection. Second, CSF1 was reportedly involved in regulating the autophagic response. The current study did not measure neuronal autophagy. Future studies are needed to explore other potential neuroprotective mechanisms underlying rh-CSF1 in the setting of HI.

## 5. Conclusions

In conclusion, intranasal administration of rh-CSF1 reduced the percentage of infarcted area, improved neurobehavioral deficits, and attenuated OS-induced neuronal degeneration and apoptosis after HI in rats. The neuronal protection of rh-CSF1 is partly mediated through the CSF1R/PLCG2/PKA/UCP2 signaling pathway. This study provides a basis for the translation of rh-CSF as a promising therapeutic strategy to patients with HIE.

## Data Availability

The data used to support the findings of this study are available from the corresponding author upon request.
